# Fine-Scale Geographical Origin of an Insect Pest Invading North America

**DOI:** 10.1371/journal.pone.0089107

**Published:** 2014-02-13

**Authors:** Takahiro Hosokawa, Naruo Nikoh, Takema Fukatsu

**Affiliations:** 1 National Institute of Advanced Industrial Science and Technology (AIST), Tsukuba, Ibaraki, Japan; 2 Department of Liberal Arts, The Open University of Japan, Chiba, Chiba, Japan; Virginia Tech, United States of America

## Abstract

Invasive species may rapidly spread throughout new areas once introduced, which may potentially lead to serious damage to local fauna and flora. Information on geographical origins, introduction routes, and biology in native regions of such invasive species is of critical importance in identifying means of transport, preventing reintroduction, and establishing control/eradication methods. The plataspid stinkbug *Megacopta cribraria*, known as kudzu bug, recently invaded North America and now has become not only an agricultural pest of soybean but also a nuisance pest. Here we investigate the geographical origin of the invasive *M. cribraria* populations. Phylogeographical analyses based on 8.7 kb mitochondrial DNA sequences of the introduced and East Asian native *Megacopta* populations identified a well-supported clade consisting of the introduced populations and *M. punctatissima* populations in the Kyushu region of Japan, which strongly suggests that the invading *M. cribraria* populations are derived from a *M. punctatissima* population in the Kyushu region. Therefore, the region is proposed as a promising source of natural enemies for biological control of the invasive pest. Based on the phylogenetic information, relationship and treatment of the two *Megacopta* species are discussed.

## Introduction

Increased world-wide trade and travel have resulted in increasing frequency of biological invasions. Invasive species may rapidly spread in new areas due to release from natural enemies, superior competitive ability, adaptation to novel environment, etc. [1], which may affect local fauna and flora and potentially cause serious damage to agriculture and fisheries [2]. In this context, information on geographical origins, introduction routes, and biology in native regions of such invasive species is critically important in identifying means of transport, preventing reintroduction, and establishing control/eradication methods [3].

The plataspid stinkbug *Megacopta cribraria* (Fabricius, 1798), known as kudzu bug or bean plataspid, is a recent invasive species in North America. This species has been known as native to Asia and Oceania, but large numbers of *M. cribraria* were discovered in northeast Georgia, USA, in 2009 [4], [5]. Now the invasive stinkbug has been reported in eight states in the southeastern USA including Georgia, South Carolina, North Carolina, Alabama, Virginia, Tennessee, Florida, and Mississippi [6] (the most recent information available at http://www.kudzubug.org), and has become not only an agricultural pest of soybean and some other leguminous crops but also a nuisance pest [4], [5], [7]. Considering the geographic distance between the native areas and the introduced area, it seems that the founders of the introduced populations have been transported by human activities, although the geographical origin of the invasive species is still elusive.

**Figure 1:**
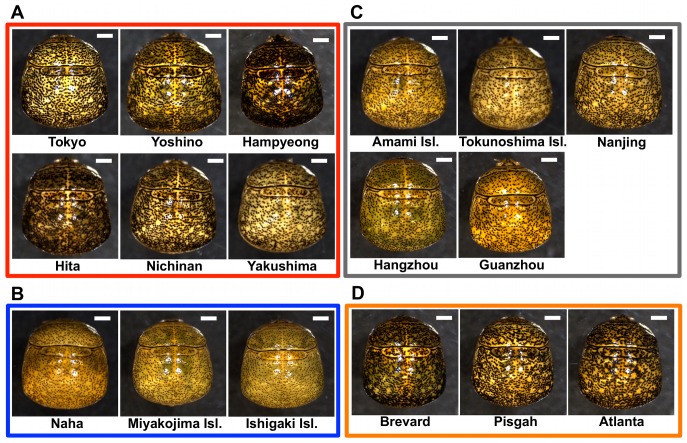
External appearance of adult females from East Asian native and North American introduced *Megacopta* populations. (A) Populations identified as *M. punctatissima*. (B) Populations identified as *M. cribraria*. (C) Populations regarded as intermediate. (D) North American introduced *M. cribraria* populations. Species identification is based on morphological characters [9], [10]. Bars show 1 mm.

*Megacopta punctatissima* (Montandon, 1896) is a closely related species to *M. cribraria* [8]. Distribution of *M. punctatissima* is restricted to East Asia, where the two *Megacopta* species show different geographical distributions: *M. punctatissima* is distributed across the three Japanese main islands of Honshu, Shikoku, and Kyushu, northern islands of the Ryukyu arc, and the Korean peninsula, while *M. cribraria* is distributed across southern Japanese islands of the Ryukyu arc, Taiwan, and China [9]. The two species are reported to be morphologically distinguishable: *M. punctatissima* exhibits larger body size, darker body color, and larger indentation size on the dorsal cuticle than *M. cribraria* [10], [11]. However, these characters may be considerably variable within and between the species, and some taxonomists regarded *M. punctatissima* as a synonym of *M. cribraria* [8] (see Fig. 1). While the North American invasive stinkbug has been identified as *M. cribraria*, molecular studies are needed to clarify the relationship among these ecologically important *Megacopta* species and populations [4], [7], [12]–[14].

Here, in order to clarify the geographical origin of the North American invasive stinkbug and its relationship to *M. cribraria* and *M. punctatissima*, we performed a phylogeographical analysis of native and invasive *Megacopta* populations on the basis of 8.7 kb mitochondrial DNA sequences.

## Materials and Methods

### Insect Samples

Adult insects of *M. cribraria* and *M. punctatissima* were collected from 50 East Asian native populations in Japan, South Korea, China, and Vietnam, and three North American introduced populations in USA. The locations are not privately-owned or protected in any way. There is no specific permission required for collecting *Megacopta* stinkbugs since these insects are quite common in the areas, and also are neither an endangered nor a protected species. Species identification was based on morphological characters [10], [11]. *Coptosoma parvipictum* was used as outgroup. Sample information is listed in Table S1.

### DNA Extraction, PCR, and Sequencing

DNA was extracted from the abdomen of each insect using QIAamp DNA Mini Kit (QIAGEN). We designed PCR primers (Table S2) to amplify mitochondrial DNA fragments with reference to a mitochondrial genome sequence of *M. cribraria* from the introduced population (accession no. JF288758) [13]. The 1.0–1.4 kb PCR products were purified, directly subjected to cycle sequencing reactions with ABI PRISM BigDye Terminator v3.1 (Applied Biosystems), and analyzed by ABI PRISM 3130×l Genetic Analyzer (Applied Biosystems).

### Phylogenetic Analyses

Multiple alignments of the gene sequences were generated using the program MAFFT 5 [15]. The aligned sequences were divided into five partitions, three codon positions in protein coding genes, tRNA genes, and non-coding regions. The program JMODELTEST [16] was used for selecting best-fit models of nucleotide substitution. Phylogenetic analyses were conducted by Bayesian (BA), maximum-likelihood (ML), and neighbor-joining (NJ) methods using the programs MrBayes 3.1.2 [17], RAxML version 7.2.1 [18], and PAUP* [19], respectively. Posterior probabilities were calculated for each node and used for statistical evaluation in BA. Bootstrap tests were conducted by 1,000 resamplings for ML and NJ.

### Nucleotide Sequence Accession Numbers

The nucleotide sequences reported in this study have been deposited in the DDBJ/EMBL/GenBank databases under accession numbers AB872048 to AB872101.

## Results and Discussion

### Origin of the North American Invasive Stinkbug

**Figure 2:**
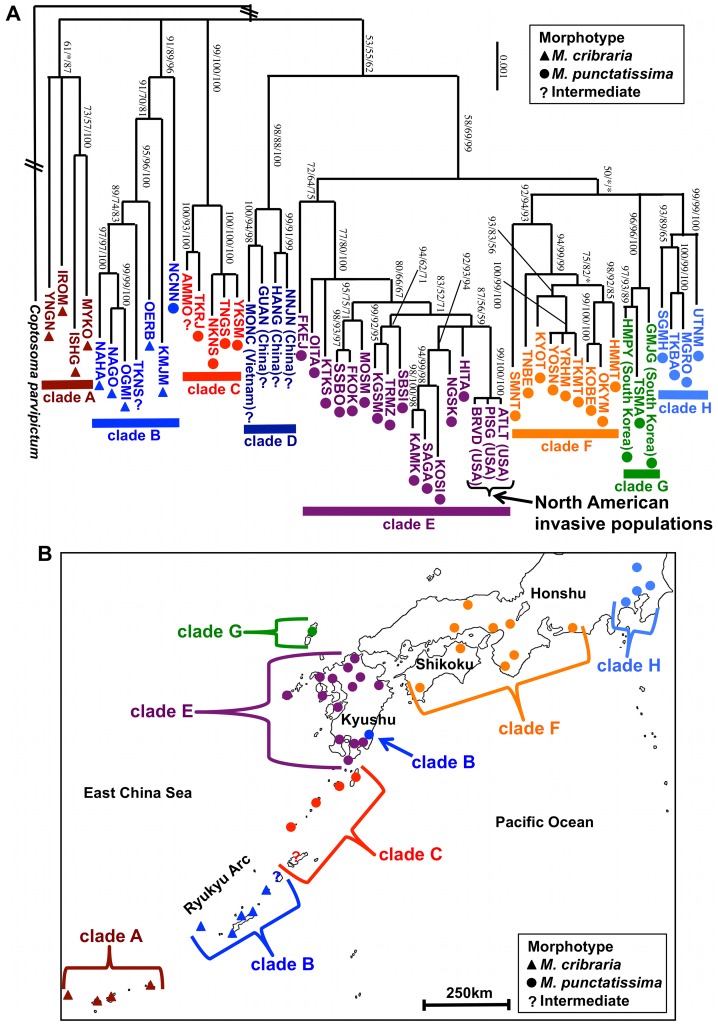
Phylogeography of *Megacopta* stinkbugs. (A) A Bayesian phylogeny of East Asian native and North American introduced *Megacopta* populations inferred from 8,552 aligned nucleotide sites of mitochondrial DNA sequences. Posterior probability of the Bayesian analysis, bootstrap probability of the maximum-likelihood analysis, and bootstrap probability of neighbor-joining analysis are indicated on each node, wherein asterisks indicate the statistical values lower than 50%. Triangles, circles, and question marks beside the sample codes indicate morphological identification of the populations as *M. cribraria*, *M. punctatissima*, and intermediate, respectively [9], [10]. Clades A–H are indicated below the sample codes in different colors. (B) Geographical locations of Japanese native *Megacopta* populations examined in this study. Clades A–H and morphotypes are indicated as in Fig. 2A.

Figure 2A shows the phylogenetic relationship of *M. cribraria* and *M. punctatissima* in their presumed native range in East Asia, wherein eight well-supported clades A–H were identified. Phylogeographical analyses of the native *Megacopta* populations revealed that geographically close populations tended to be placed in the same clades with an exception, Nichinan population (NCNN), which was embraced in the clade B but geographically distant from the other clade-B populations (Fig. 2B and Fig. S1). Notably, the three introduced *M. cribraria* populations were included in the clade E (Fig. 2A). All the other clade-E populations were located in the Kyushu region of Japan (Fig. 2B), and have been identified as *M. punctatissima* [10], [11]. Although we had no chance to examine South Asian, Southeast Asian, and Oceanian populations of *M. cribraria,* on account of the relative geographical distance, it seems unlikely that these populations belong the clade E. These results strongly suggest that the invasive *M. cribraria* populations in USA are derived from a *M. punctatissima* population in the Kyushu region of Japan. Actually, morphological characters, such as body color and indentation size on the dorsal cuticle, of the introduced populations looked similar to those of *M. punctatissima* rather than *M. cribraria* (Fig. 1). Since no mitochondrial haplotype completely or nearly identical to that of the introduced *M. cribraria* populations has been identified, it has not yet been determined which population in the Kyushu region is the origin of invasion. More comprehensive sampling and investigation of population genetic structure in the Kyushu region are needed.

In this study, we identified a single mitochondrial haplotype, represented by identical 8,687 bp mitochondrial DNA sequences, from the three introduced *M. cribraria* populations in USA. In previous studies, identical mitochondrial DNA sequences were obtained from 269 individuals collected across a wide spatial and temporal range in the southeastern USA [7], [13]. Notably, the sequence reported in the previous studies (accession no. JF288758) exhibits a slight discrepancy (3/8,687 aligned nucleotide sites) with the sequence determined in this study. The small differences are probably due to either small genetic differences between the insect samples or method-dependent consistent sequencing errors.

To date, no natural enemies that effectively suppress the introduced *M. cribraria* populations in USA have been identified, and thus importation of exotic natural enemies, such as egg parasitoid wasps, from the native range may offer a possible biological control means against the invasive pest insect [7]. Here we suggest the Kyushu region as a promising source of such natural enemies, considering their potential local adaptation against the target pest population [3].

### Relationships between *M. cribraria* and *M. punctatissima*

The phylogenetic analysis divided the *Megacopta* populations into eight distinct clades A–H, which largely reflected their geographic ranges (Fig. 2). Morphologically, the clades A and B were regarded as *M. cribraria* whereas the clades C, E, F, G and H were classified to *M. punctattisima* (Fig. 1). Notably, some populations, like Chinese populations constituting the clade D and also Amami and Tokunoshima populations located at the geographic boundary of the clades B and C (Fig. S1), were morphologically intermediate between *M. cribraria* and *M. punctatissima* (Fig. 1). The clade B mostly consisting of *M. cribraria* populations also contained a *M. punctatissima* population (Fig. 2), which may be due to mitochondrial introgression from southern island populations to the Kyushu population via accidental migration and crossbreeding [20]. Although the phylogenetic relationship between the clades was poorly resolved, the overall phylogenetic patterns suggest that neither *M. cribraria* nor *M. punctatissima* constitutes a monophyletic group, favoring the idea that *M. cribraria* and *M. punctatissima* do not constitute distinct species but rather represent local populations of the same species with considerable genetic and phenotypic diversity. Meanwhile, we point out that grasping and understanding of the genetic and phenotypic aspects of the *Megacopta* clades are of pivotal biological importance, considering that they may exhibit different local adaptations and ecological traits such as plant adaptation and pest status [20]. How the *M. cribraria*-*M. punctatissima* complex should be treated taxonomically is entrusted to experts of stinkbug taxonomy and systematics.

## Supporting Information

Figure S1 **Geographical locations of East Asian native** ***Megacopta*** **populations examined in this study.** Several Japanese populations are omitted (see Fig. 2B). Clades A–H and morphotypes of the populations are indicated as in figure 1. Inset shows this East Asian area depicted by a red rectangle on a larger-scale map.
(TIFF)

Table S1 **Insect samples used in this study.**
(PDF)

Table S2 **Primer sets used in this study.**
(PDF)

## References

[1] Sax DF, Stachowicz JJ, Gaines SD (2005) Species invasions: insights into ecology, evolution, and biogeography. Sunderland: Sinauer. 495 p.

[2] Pimentel D, McNair S, Janecka J, Wightman J, Simmonds C, et al (2001) Economic and environmental threats of alien plant, animal, and microbe invasions. Agric Ecosyst Environ 84: 1–20.

[3] Estoup A, Guillemaud T (2010) Reconstructing routes of invasion using genetic data: why, how and so what? Mol Ecol 19: 4113–4130. 20723048 10.1111/j.1365-294X.2010.04773.x

[4] Eger JE Jr, Ames LM, Suiter DR, Jenkins TM, Rider DA, et al (2010) Occurrence of the Old World bug *Megacopta cribraria* (Fabricius) (Heteroptera: Plataspidae) in Georgia: a serious home invader and potential legume pest. Insecta Mundi 0121: 1–11.

[5] Suiter DR, Eger JE Jr, Gardner WA, Kemerait RC, All JN, et al (2010) Discovery and distribution of *Megacopta cribraria* (Hemiptera: Heteroptera: Plataspidae) in northeast Georgia. J Integrat Pest Manag 1: 1–4.

[6] Gardner WA, Peeler HB, LaForest J, Roberts PM, Sparks AN Jr, et al (2013) Confirmed distribution and occurrence of *Megacopta cribraria* (F.) (Hemiptera: Heteroptera: Plataspidae) in the Southeastern United States. J Entomol Sci 48: 118–127.

[7] Ruberson JR, Takasu K, Buntin GD, Eger JE Jr, Gardner WA, et al (2013) From Asian curiosity to eruptive American pest: *Megacopta cribraria* (Hemiptera: Plataspidae) and prospects for its biological control. Appl Entomol Zool 48: 3–13.

[8] Yang WI (1934) Revision of Chinese Plataspidae. Bull Fan Inst Biol 5: 137–236.

[9] Ishikawa T, Takai M, Yasunaga T (2012) A field guide to Japanese bugs. Terrestrial Heteropterans. Vol. 3. Tokyo: Zenkoku Noson Kyoiku Kyokai Publishing Co., Ltd. (in Japanese). 576 p.

[10] Montandon AL (1896) Plataspidinae. Nouvelle série d’études et descriptions. Ann Soc Entomol Belg 40: 86–134.

[11] Tomokuni M, Yasunaga T, Takai M, Yamashita I, Kawamura M, et al.. (1993) A field guide to Japanese bugs. Terrestrial heteropterans. Tokyo: Zenkoku Noson Kyoiku Kyokai Publishing Co., Ltd. (in Japanese). 380 p.

[12] Jenkins TM, Suiter D, Eger J, Ames L, Buntin D, et al (2010) The preliminary genetics of an invasive true bug from the old world: implications for the new world. J Entomol Sci 45: 1–2.

[13] Jenkins TM, Eaton TD (2011) Population genetic baseline of the first plataspid stink bug symbiosis (Hemiptera: Heteroptera: Plataspidae) reported in North America. Insects 2: 264–272. 26467727 10.3390/insects2030264 PMC4553543

[14] Brown AMV, Huynh LY, Bolender CM, Nelson KG, McCutcheon JP (2013) Population genomics of a symbiont in the early stages of a pest invasion. Mol Ecol Article first published online: 11 JUL 2013 DOI: 10.1111/mec.12366. 10.1111/mec.12366 23841878

[15] Katoh K, Kuma K, Toh H, Miyata T (2005) MAFFT version 5: improvement in accuracy of multiple sequence alignment. Nucleic Acids Res 33: 511–518. 15661851 10.1093/nar/gki198 PMC548345

[16] Posada D (2008) jModelTest: Phylogenetic model averaging. Mol Biol Evol 25: 1253–1256. 18397919 10.1093/molbev/msn083

[17] Ronquist F, Huelsenbeck JP (2003) MrBayes 3: Bayesian phylogenetic inference under mixed models. Bioinformatics 19: 1572–1574. 12912839 10.1093/bioinformatics/btg180

[18] Stamatakis A (2006) RAxML-VI-HPC: maximum likelihood-based phylogenetic analyses with thousands of taxa and mixed models. Bioinformatics 22: 2688–2690. 16928733 10.1093/bioinformatics/btl446

[19] Swofford DL (2003) PAUP*. Phylogenetic Analysis Using Parsimony (*and Other Methods). Version 4. Sinauer Associates, Sunderland, Massachusetts.

[20] Hosokawa T, Kikuchi Y, Shimada M, Fukatsu T (2007) Obligate symbiont involved in pest status of host insect. Proc R Soc B 274: 1979–1984. 10.1098/rspb.2007.0620 PMC2275188 17567556

