# Supplementary figures and images for: Fine-Scale Geographical Origin of an Insect Pest Invading North America
Source: PLoS One. 2014 Feb 13;9(2):e89107. doi: 10.1371/journal.pone.0089107 (PMC3923857; doi:10.1371/journal.pone.0089107)

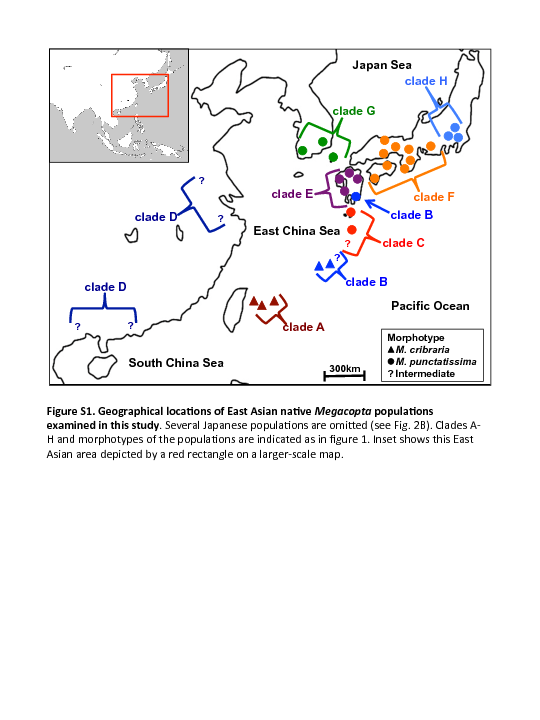

Supplement: Figure S1 — Geographical locations of East Asian native Megacopta populations examined in this study. Several Japanese populations are omitted (see Fig. 2B). Clades A–H and morphotypes of the populations are indicated as in figure 1. Inset shows this East Asian area depicted by a red rectangle on a larger-scale map. (TIFF) [file pone.0089107.s001.tiff]
