# Supplementary material for: Fine-Scale Geographical Origin of an Insect Pest Invading North America
Source: PLoS One. 2014 Feb 13;9(2):e89107. doi: 10.1371/journal.pone.0089107 (PMC3923857; doi:10.1371/journal.pone.0089107)
Supplement: Table S1 — Insect samples used in this study. (PDF) [file pone.0089107.s002.pdf]

Table S1. Insect samples used in this study.

Sample code	Morpho-type[1]	Collection site[2]	Collector	Collection year	Accession number[3]
UTNM	Mp	Utsunomiya, Tochigi, J	S. Tsuboyama	2012	AB872048
TKBA	Mp	Tsukuba, Ibaraki, J	T. H.	2003	AB872049
MGRO	Mp	Meguro, Tokyo, J	N. Kaiwa	2009	AB872050
SGMH	Mp	Sagamihara, Kanagawa, J	T. F.	2012	AB872051
HMMT	Mp	Hamamatsu, Shizuoka, J	M. Hironaka	2011	AB872052
KYOT	Mp	Kyoto, Kyoto, J	S. Kada	2009	AB872053
YOSN	Mp	Yoshino, Nara, J	K. Tsuji	2010	AB872054
TNBE	Mp	Tanabe, Wakayama, J	T. H.	2008	AB872055
KOBE	Mp	Kobe, Hyogo, J	T. H.	2008	AB872056
YRHM	Mp	Yurihama, Tottori, J	N. Tsurusaki	2009	AB872057
OKYM	Mp	Okayama, Okayama, J	C. Himuro	2009	AB872058
TKMT	Mp	Takamatsu, Kagawa, J	Y. Kikuchi	2009	AB872059
SMNT	Mp	Shimanto, Kochi, J	Y. Kikuchi	2009	AB872060
KTKS	Mp	Kitakyushu, Fukuoka, J	M. Baba	2011	AB872061
FKOK	Mp	Fukuoka, Fukuoka, J	G. Sakurai	2006	AB872062
SAGA	Mp	Saga, Saga, J	H. Mukai	2011	AB872063
TSMA	Mp	Tsushima, Nagasaki, J	T. H.	2009	AB872064
SSBO	Mp	Sasebo, Nagasaki, J	M. Tokuda	2012	AB872065
NGSK	Mp	Nagasaki, Nagasaki, J	S. Ohba	2012	AB872066
FKEJ	Mp	Fukuejima, Nagasaki, J	H. Hirayama	2009	AB872067
HITA	Mp	Hita, Oita, J	T. Yanagi	2011	AB872068
OITA	Mp	Oita, Oita, J	K. Ito	2012	AB872069
KOSI	Mp	Koshi, Kumamoto, J	Y. Kikuchi	2009	AB872070
KAMK	Mp	Kamiamakusa, Kumamoto, J	N. Endo	2012	AB872071
NCNN	Mp	Nichinan, Miyazaki, J	T. H.	2008	AB872072
KGSM	Mp	Kagoshima, Kagoshima, J	M. Tokuda	2012	AB872073
SBSI	Mp	Shibushi, Kagoshima, J	M. Tokuda	2012	AB872074
TRMZ	Mp	Tarumizu, Kagoshima, J	M. Tokuda	2012	AB872075
MOSM	Mp	Minamiosumi, Kagoshima, J	M. Tokuda	2012	AB872076
TNGS	Mp	Tanegashima, Kagoshima, J	T. H.	2009	AB872077
YKSM	Mp	Yakushima, Kagoshima, J	T. F.	2008	AB872078
NKNS	Mp	Nakanoshima, Kagoshima, J	M. Tanahashi	2011	AB872079
TKRJ	Mp	Takarajima, Kagoshima, J	M. Tanahashi	2011	AB872080
AMMO	?	Amami-oshima, Kagoshima, J	T. H.	2009	AB872081
TKNS	?	Tokunoshima, Kagoshima, J	T. H.	2009	AB872082
OERB	Mc	Okinoerabujima, Kagoshima, J	T. H.	2011	AB872083
OGMI	Mc	Ogimi, Okinawa, J	R. Ukuda	2010	AB872084
NAGO	Mc	Nago, Okinawa, J	T. H.	2009	AB872085
NAHA	Mc	Naha, Okinawa, J	S. Ohno	2004	AB872086
KMJM	Mc	Kumejima, Okinawa, J	T. H.	2010	AB872087
MYKO	Mc	Miyakojima, Okinawa, J	T. H.	2008	AB872088
ISHG	Mc	Ishigakijima, Okinawa, J	T. H.	2009	AB872089
IROM	Mc	Iriomotejima, Okinawa, J	T. H.	2008	AB872090
YNGN	Mc	Yonagunijima, Okinawa, J	T. H.	2011	AB872091
GMJG	Mc	Geumjeong, Busan, K	Y.-H. Jo	2011	AB872092
HMPY	Mc	Hampyeong, Jeollanam, K	Y.-H. Jo	2012	AB872093

Continued on next page

Table S1. Continued

Sample code	Morpho-types[1]	Collection site[2]	Collector	Collection year	Accession number[3]
NNJN	?	Nanjing, Jiangsu, C	H.-L. Ji	2012	AB872094
HANG	?	Hangzhou, Zhejiang, C	X.-L. Bing	2012	AB872095
GUAN	?	Guangzhou, Guangdong, C	H.-Y. Chen	2012	AB872096
MONC	?	Mong Cai, Quang Ninh, V	S. Ohno, D. Haraguchi, K. Kijima	2007	AB872097
BRVD	IP	Brevard, North Carolina, U	L. Huynh	2011	AB872098
PISG	IP	Pisgah, North Carolina, U	L. Huynh	2011	AB872099
ATLT	IP	Atlanta, Georgia, U	N. M. Gerardo	2012	AB872100
CPAR	Cp	Kanzaki, Saga, J	T. H.	2007	AB872101

[1]Species identification is based on morphological characters [Montandon 1896, Tomokuni et al. 1993]: Mp, *M. punctatissima*; Mc, *M. cribraria*; ?, intermediate; Cp, *Coptosoma parvipictum*; IP, the North American introduced population.
[2]J, Japan; K, Korea; C, China; V, Vietnam; U, USA.
[3]A total of about 8.7 kb mitochondrial DNA sequence.
