# Supplementary material for: Fine-Scale Geographical Origin of an Insect Pest Invading North America
Source: PLoS One. 2014 Feb 13;9(2):e89107. doi: 10.1371/journal.pone.0089107 (PMC3923857; doi:10.1371/journal.pone.0089107)
Supplement: Table S2 — Primer sets used in this study. (PDF) [file pone.0089107.s003.pdf]

Table S2. Primer sets used in this study.

Primer sets (5'-3')	Annealing temperature	Genes in amplified fragment	References
179F[1], AGCTAATAGGTTCATACCCTA<br>1452R[1], GTTCAATAGATAAAGTGGCTG	51°C	Partial $tRNA^{Met}$, *ND2*, $tRNA^{Trp}$, $tRNA^{Cys}$, partial $tRNA^{Tyr}$	This study
Mc1239F[2], AACATATTTGTAGCGAAAGCT<br>Mc2673R[2], TTTAGGGTTTAAACTAAGTCC	50°C	Partial $tRNA^{Trp}$, $tRNA^{Cys}$, $tRNA^{Tyr}$, partial *COI*	This study
Cp1239F[3], CTGTCAATTTACCTAAAAGCT<br>Cp2673R[3], TTTAGGGTTTATTGTAAGTCC	50°C	Partial $tRNA^{Trp}$, $tRNA^{Cys}$, $tRNA^{Tyr}$, partial *COI*	This study
CO2F[1], CTATTCACAATCGGAGGACTAA<br>CO2R[1], GGTTTAAGAGACCAATGCT	51°C	Partial *COI*, $tRNA^{Leu}$, *COII*, partial $tRNA^{Lys}$	Jenkins & Eaton (2011)
3652F[1], GGCTAATCACTCATTTATACC<br>4873R[1], ATCTCGTCATCATTGGTATAT	50°C	Partial *COII*, $tRNA^{Lys}$, $tRNA^{Asp}$, *ATP8*, *ATP6*, partial *COIII*	This study
4717F[1], CACTTAGTAGATTACAGACCA<br>6008R[1], CTAATTTTTAGGTCGAAACTAAATG	51°C	Partial *COIII*, $tRNA^{Gly}$, *ND3*, $tRNA^{Ala}$, partial $tRNA^{Arg}$	This study
5853F[1], ATATCATGAATGAAAAAACGG<br>7067R[1], AGACTCTCAAGATATTCGT	47°C	Partial *ND3*, $tRNA^{Ala}$, $tRNA^{Arg}$, $tRNA^{Asn}$, $tRNA^{Ser}$, $tRNA^{Glu}$, $tRNA^{Phe}$, partial *ND5*	This study
Mc6911F[2], ACCCCCCATAGTTATATATTC<br>Mc8072R[2], AGAATAATAATTTGTGGTATTATAGG	50°C	Partial *ND5*, partial $tRNA^{His}$	This study
Cp6912F[3], CCACTCAAAGTTATATATTCA<br>Cp8072R[3], GATAATTTGTGGTGTTATTGG	48°C	Partial *ND5*, partial $tRNA^{His}$	This study
7891F[1], AATTTAATGAATAAACCTCCCA<br>9003R[1], CTTGCTTTTTATACTTTATTTGC	49°C	Partial *ND5*, $tRNA^{His}$, partial *ND4*	This study

[1]Primers for both *Megacopta* species and *Coptosoma parvipictum*. [2]Primers for *Megacopta* species. [3]Primers for *C. parvipictum*.
